# Hypoxia-induced soluble CD137 in malignant cells blocks CD137L-costimulation as an immune escape mechanism

**DOI:** 10.1186/2051-1426-2-S3-P218

**Published:** 2014-11-06

**Authors:** Sara Labiano, Asis Palazon, Elixabet Bolaños-Mateo, Arantza Azpilicueta, Alfonso Rodriguez, Aizea Morales-Kastresana, Elena Marin, Alfonso Gurpide, Maria Rodriguez-Ruiz, M Angela Aznar, Maria Jure-Kunkel, Ignacio Melero

**Affiliations:** 1 Immunology and Immunotherapy, Center for Applied Medical Research, Pamplona, Spain; 2 Oncology, CUN, Pamplona, Spain; 3 Oncology Drug Discovery division, Bristol-Myers Squibb Pharmaceutical Research Institute, Princeton, NJ, USA

## 

Hypoxia is a common feature in solid tumors that has been implicated in immune-evasion. Previous studies from our group have shown that hypoxia up-regulates the co-stimulatory receptor CD137 on activated T lymphocytes and on vascular endothelial cells. In this study, we show that exposure of mouse and human tumor cell lines to hypoxic conditions (1% O_2_) promotes CD137 transcription. However, the resulting mRNA is predominantly an alternatively spliced form that encodes for a soluble variant, lacking the transmembrane domain. Accordingly, soluble CD137 (sCD137) is detectable by ELISA in the supernatant of hypoxia-exposed cell lines and in the serum of tumor-bearing mice. sCD137, as secreted by tumor cells, is able to bind to CD137-Ligand (CD137L). Our studies on primed T lymphocytes in co-culture with stable transfectants for CD137L demonstrate that tumor-secreted sCD137 prevents co-stimulation of T lymphocytes. Such an effect results from preventing the interaction of CD137L with the transmembrane forms of CD137 expressed on T lymphocytes undergoing activation. This mechanism is interpreted as a molecular strategy deployed by tumors to repress lymphocyte co-stimulation via CD137/CD137L.

